# The Epidemiology of Influenza Virus Infection and Group A Streptococcal Pharyngitis in Children Between 2011 and 2018 in an Outpatient Pediatric Clinic

**DOI:** 10.7759/cureus.33492

**Published:** 2023-01-07

**Authors:** Ismail Yildiz, Erdem Gonullu, Ahmet Soysal, Cevat Naci Oner, Metin Karabocuoglu

**Affiliations:** 1 Pediatrics, Yalova University, Faculty of Medicine, Yalova, TUR; 2 Pediatrics, Istanbul Health and Technology University, Faculty of Medicine, İstanbul, TUR; 3 Pediatrics, Memorial Ataşehir Hospital, İstanbul, TUR; 4 Pediatrics, Biruni University, İstanbul, TUR

**Keywords:** children, seasonal distribution, frequency, streptococcus pyogenes, influenza virus

## Abstract

Background

The frequency of influenza virus infections and group A beta-hemolytic streptococcus (GAS) pharyngitis varies according to populations. We aimed to investigate the frequency of influenza virus and streptococcal pharyngitis infections in a pediatric outpatient cohort with many pediatric admissions in Istanbul.

Materials and methods

Children with upper respiratory tract infection (URTI) symptoms between 2011 and 2018 who underwent rapid diagnostic tests for influenza virus or streptococcal infection were evaluated retrospectively.

Results

The total number of pediatric cases admitted between 2011 and 2018 was 185,228, of which 119,928 were under five years old and 66,300 were children over five years old. The mean frequency of the influenza virus was 1,283 per 100,000 children, and the mean frequency of streptococcal pharyngitis was 1,764 per 100,000 children. The frequency of influenza has increased over the years. The frequency of streptococcal infection is higher in children over five years of age, and its frequency has decreased in this group.

Conclusions

The frequency of influenza virus infection and GAS pharyngitis varies according to years and seasons. Winter and spring were the seasons with the most frequent positive influenza virus and GAS pharyngitis. Although influenza frequency increased annually, this phenomenon was not observed in the frequency of GAS pharyngitis.

## Introduction

Upper respiratory tract infections (URTIs) are essential for referrals to outpatient pediatric clinics. While children have an average of 6-8 URTIs per year, this may vary according to age groups. URTIs can be seen 2-8 times a year in younger age groups and 3-6 times a year in older children [1,2]. Although viruses are the most common cause of URTIs in children, etiological factors may vary according to age groups [3]. The frequency of seasonal influenza virus infections varies according to age in the range of 0.3%-9.8% between zero and 14 years of age. Although influenza virus infection may occur in every age group, the clinical course can be more severe and complicated in younger children [4].

The common etiological agent of bacterial pharyngitis is *Streptococcus pyogenes* (or group A beta-hemolytic streptococcus {GAS}). GAS is responsible for 15%-30% of pharyngitis cases in children aged 5-15 years [5].

Nowadays, the use of rapid antigen tests in the diagnosis of cases with URTI symptoms has increased. Planning the treatment according to the etiological agent provides a significant advantage in patients diagnosed through these tests. Furthermore, the rational application of drugs for the causative agent prevents unnecessary antibiotic use and resistance [6].

In this study, the frequency of influenza virus infection and GAS pharyngitis was investigated concerning age, using rapid diagnostic tests in children between 2011 and 2018.

## Materials and methods

This retrospective study was performed at Memorial Ataşehir Hospital and Memorial Şişli Hospital, located in two different districts of Istanbul (population > 15 million, approximately 20% of Turkey’s inhabitants), Turkey. The cases were children aged from one month to 18 years admitted to our study hospitals’ pediatric outpatient and emergency room units with signs of URTI between January 1, 2011, and December 31, 2018, who were administered rapid diagnostic tests for influenza virus and streptococcal swabs to clarify the etiology. One Step Rapid Test (Healgen Scientific LLC, Houston, TX, USA) and Strep A Rapid Test (Healgen Scientific LLC, Houston, TX, USA) were used as rapid tests for influenza A or B infection and as a quick test for GAS pharyngitis, respectively.

The study population was divided into two groups: patients under five years and older than five years old. The number of patients admitted was established by protocol numbers that also included the identification number of each patient via hospital databases. We evaluated the results of cases that were applied more than once weekly as a single application. Repeat admissions for the same diagnosis detected by patient identification numbers were excluded.

Frequencies were calculated as follows: frequency of influenza virus infection or GAS pharyngitis per 100,000 population = number of positive cases per year/size of admission population per year × 100,000. Therefore, the annual frequency rates of influenza and GAS pharyngitis were shown as separate graphs. The study was approved by the Memorial Şişli Hospital Ethics Committee on August 29, 2019 (approval number: 2019/004).

Statistical analysis

Data were entered into a pocket spreadsheet program (Microsoft Excel {Microsoft® Corp., Redmond, WA, USA} for Mac {2018}), and statistical analyses were carried out with Jamovi 1.8 (The jamovi project, Sydney, Australia {2021}) (retrieved from https://www.jamovi.org). Descriptive data have been reported, and frequency values have been analyzed by age and years. In addition, categorical variants were analyzed using the chi-square test. To measure the linear correlation between the study period (years) and the frequencies of GAS pharyngitis and influenza infection, we used the Pearson correlation coefficient (r). A significance level of p < 0.05 was considered statistically significant for statistical comparisons.

## Results

A total of 185,228 children were admitted to the study hospitals between 2011 and 2018. Among them, 119,928 (65%) children were under five years of age, and 66,300 (35%) were ≥5 years old. The yearly admission number gradually increased with time, ranging from 15,361 in 2011 to 29,698 in 2018 (p < 0.05).

**Figure 1:**
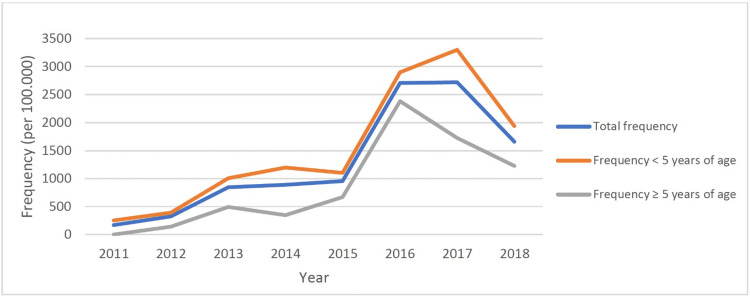
Frequency of influenza according to years and age groups

During the study period from 2011 to 2018, 2,664 children were diagnosed with influenza (mean: 333 children/year; range: 26-772 children/year). The annual frequencies of influenza in the pediatric age group are shown in Table 1, exhibiting significant increasing trends (p = 0.011; r = 0.827) from 169 to 1,660 per 100,000 children admitted. Moreover, the annual frequencies of influenza in children under five years of age are shown in Table 1, with significant increasing trends (p = 0.010; r = 0.836) from 251 to 1,934 per 100,000 admissions. An increase was also observed in the annual influenza frequency in children aged ≥5 years (p = 0.021; r = 0.785) from 141 to 1,228 per 100,000 admissions during the study period (Figure 1).

**Table 1:** The annual frequency of influenza by years and age groups *The difference between influenza virus infection in children <5 years old and ≥5 years old NA: not applicable

Years	Total number of admissions	Total number of influenza cases	Influenza frequency per 100,000 total	Total number of admissions for <5 years old	Number of influenza cases for <5 years old	Influenza frequency per 100,000 for <5 years old	Total number of admissions for ≥5 years old	Number of influenza cases for ≥5 years old	Influenza frequency per 100,000 for ≥5 years old	P-value*
2011	15,361	26	169.3	10,323	26	251.9	5,038	0	0	NA
2012	16,490	54	327.5	11,844	46	388.4	5,646	8	141.7	0.006
2013	21,386	180	841.7	14,489	146	1,007.7	6,897	34	493	<0.001
2014	24,397	216	885.4	15,491	185	1,194.2	8,906	31	348.1	<0.001
2015	23,818	227	953.1	15,613	172	1,101.6	8,205	55	670.3	0.001
2016	25,693	696	2,708.9	16,122	468	2,902.9	9,571	228	2,382.2	0.013
2017	28,385	772	2,719.7	17,906	591	3,300.6	10,479	181	1,727.3	<0.001
2018	29,698	493	1,660	18,140	351	1,934.9	11,558	142	1,228.6	<0.001

**Figure 2:**
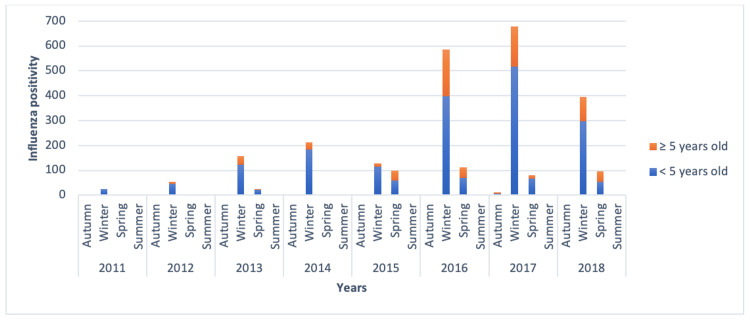
Influenza virus positivity in children according to seasons over the years

A comparison of the annual influenza frequency between the age groups revealed that the annual frequency of influenza was higher in children under five years of age than in children aged ≥5 years during the study period from 2011 to 2018. The influenza virus is detected primarily in winter in all age groups but also in early spring. The distribution of influenza cases according to the seasons concerning study years and age groups is shown in Figure 2.

**Figure 3:**
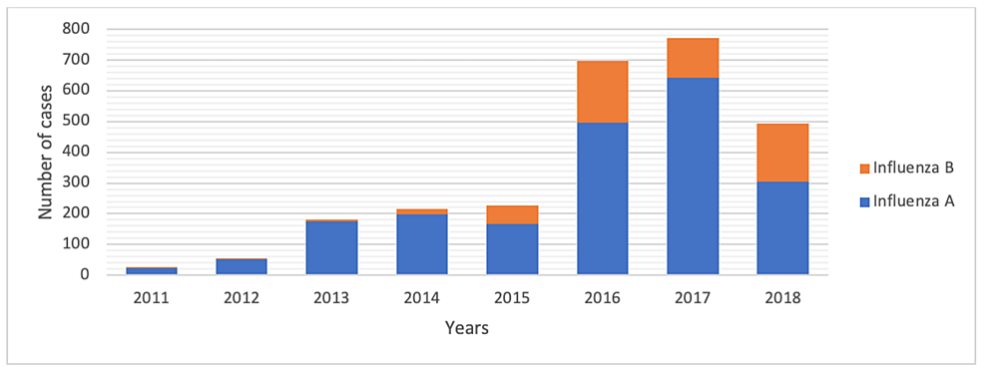
Influenza A and influenza B virus positivity in children according to years

Of the 2,664 influenza virus-positive cases detected during the study, 2,062 (77.4%) were influenza A-positive, and 602 (22.6%) were influenza B-positive. Influenza A virus positivity was detected more frequently in all years (Figure 3).

During the study period from 2011 to 2018, 3,191 children were diagnosed with GAS pharyngitis (mean: 399 children/year; range: 263-643 children/year). The annual frequencies of GAS pharyngitis in the pediatric age group are shown in Table 2.

**Table 2:** Annual frequency of group A beta-hemolytic streptococcus (GAS) pharyngitis by years and age groups *The difference between group A beta-hemolytic streptococcus (GAS) pharyngitis in children <5 years old and ≥5 years old

Years	Total number of admissions	Total number of GAS pharyngitis cases	The annual frequency of GAS pharyngitis per 100,000 total	Total number of admissions for <5 years old	Number of GAS pharyngitis cases for <5 years old	The annual frequency of GAS pharyngitis per 100,000 for <5 years old	Total number of admissions for ≥5 years old	Number of GAS pharyngitis cases for ≥5 years old	The annual frequency of GAS pharyngitis per 100,000 for ≥5 years old	P-value*
2011	15,361	431	2,805.8	10,323	133	1,288.4	5,038	298	5915	<0.001
2012	16,490	263	1,594.9	11,844	56	472.8	5,646	207	3,666.3	<0.001
2013	21,386	282	1,318.6	14,489	102	704	6,897	180	2,609.8	<0.001
2014	24,397	375	1,537.1	15,491	173	1,116.8	8,906	202	2,268.1	<0.001
2015	23,818	288	1,209.2	15,613	128	819.8	8,205	160	1,950	<0.001
2016	25,693	643	2,502.6	16,122	407	2,524.5	9,571	236	2,465.8	0.771
2017	28,385	510	1,796.7	17,906	343	1,915.6	10,479	167	1,593.7	0.049
2018	29,698	399	1,343.5	18,140	260	1,433.3	11,558	139	1,202.6	0.092

**Figure 4:**
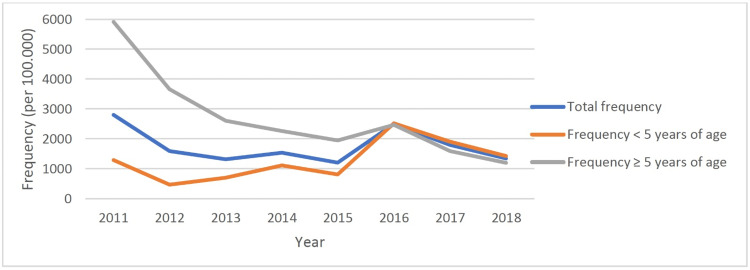
Frequency of group A beta-hemolytic streptococcus (GAS) pharyngitis according to years and age groups

The annual frequencies of GAS pharyngitis in the pediatric age group did not change during the study period (p = 0.47; r = -0.299). Moreover, the annual frequencies of GAS pharyngitis in children under five did not change significantly during the study period (p = 0.134; r = 0.578). However, the annual frequencies of GAS pharyngitis in children aged ≥5 years decreased significantly during the study period (p = 0.006; r = -0.863). A comparison of the annual frequencies of GAS pharyngitis between the age groups revealed that the annual frequency of GAS pharyngitis was higher in children aged ≥5 years than in children under the age of five over the entire study period. The distribution of GAS pharyngitis cases according to seasons concerning study period and age groups is shown in Figure 4.

**Figure 5:**
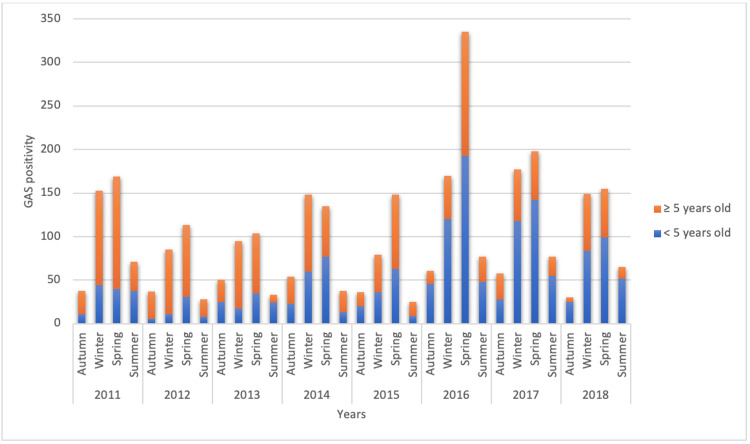
Group A beta-hemolytic streptococcus (GAS) pharyngitis in children according to seasons over the years

GAS pharyngitis is detected primarily in all age groups in the spring and winter seasons (Figure 5).

The mean rate of detection of influenza positivity was 12.2%, and the mean rate of detection of GAS positivity was 18.2% during the study period.

## Discussion

This study summarizes the annual frequency of influenza virus infection and GAS pharyngitis in children for an eight-year retrospective observational period. It is shown that the annual frequency of influenza virus infections in children ranges from 169 to 2,719 per 100,000 admissions (average: 1,283 per 100,000). The frequency of influenza infections in children may vary according to age. In the present study, the frequency of influenza virus infection was higher in children under five years of age than in children above the age of five years for all study years. The mean frequency rate of influenza virus infection varied from 354 to 9,784 per 100,000 children under four years and 315 to 8,915 per 100,000 children over four years old, according to a multicentric European study [4]. In the present study, the mean frequency of influenza virus infections between 2011 and 2018 was 1,283 per 100,000 children admitted, and the mean influenza frequency in patients under five and ≥5 years of age was 1,510 and 874 per 100,000, respectively. These differences may be explained by identifying the source of fever in small children leading to doctors demanding more diagnostic tests. In addition, the onset and peak times of influenza virus infections may vary according to regions and years [7-11]. In this study, influenza virus infections are often seen in autumn and reach a peak in winter seasons.

The second finding of this study is the detection of GAS pharyngitis in children under five years of age. Identifying and treating GAS pharyngitis are essential in the childhood age group because serious suppurative and nonsuppurative complications can occur if they remain untreated [12]. One of the most critical nonsuppurative complications is acute rheumatic fever (ARF). Turkey is in a medium/high-risk group for ARF in children with an annual frequency of ARF of >2 per 100,000 children [13,14]. Therefore, the diagnosis and treatment of GAS pharyngitis remain essential. Most of the classical literature accepted that GAS pharyngitis mainly occurs in children at 5-15 years of age [15]. This study found that the frequency of GAS pharyngitis in the pediatric age group was 1,764 per 100,000 admissions. On the other hand, the mean GAS pharyngitis frequency in under five years old (1,284 per 100,000 children admitted) was found to be less than half the frequency seen in children above five years (2,709 per 100,000 children admitted). Therefore, pediatricians should test for GAS pharyngitis in febrile young children, even those under the age of five years. While throat culture was the gold standard diagnostic method for GAS pharyngitis, rapid streptococcal antigen tests can obtain faster results with higher sensitivity and specificity. In a review evaluating the role of rapid antigen tests in diagnosing GAS pharyngitis in children, these tests were found to be sensitive at 85.6% (95% confidence interval {CI}: 83.3%-87.6%) and specific at 95.4% (95% CI: 94.5%-96.2%) [16]. Antibiotic treatment can be started when the rapid streptococcal antigen test is positive. Still, when the test is negative, throat culture, which is the gold standard for definitive diagnosis, should be performed in children with URTI symptoms. In this study, only cases with positive rapid antigen tests were evaluated. One of the present study’s limitations is the absence of a throat culture evaluation. Wu et al. found the frequency of culture-positive GAS pharyngitis in children to be 16,527 per 100,000 between 2012 and 2014 [17]. In the present study, the mean frequency of strep A-positive GAS pharyngitis between 2011 and 2018 was 1,764 per 100,000 children. In this study, GAS pharyngitis was encountered in all seasons, but it was more common in winter and spring.

Although the prominent features of this data were evaluated together with the frequency of influenza virus infection and GAS pharyngitis according to years and seasons, the weakest aspect of the study was the difference in frequency according to years depending on the supply and use of influenza and rapid streptococcal test kits. Rapid tests were used to detect GAS in a throat swab and influenza virus in a nasopharyngeal swab. Since the sensitivity of rapid diagnostic tests is approximately 80%, it is estimated that the actual frequency underlying this study may be 20% higher. This uncertainty was one of the limitations of the study. For this purpose, more reliable data should be obtained by conducting studies with prospectively planned cases followed up according to years.

In the study of Taymaz et al., it was reported that antibiotics were prescribed in approximately one-fifth of the cases with positive influenza rapid antigen tests [18]. Therefore, although it is seen that antibiotics are prescribed for individuals with positive influenza tests in studies from Turkey, it should be encouraged to reduce unnecessary antibiotic use within the scope of rational antibiotic use in childhood.

In our study, the most common period of influenza A was the early winter period, and influenza B was between the early winter and early spring (between January and March). Although this finding is in line with the weekly influenza report announced by the Turkish health authority, the frequency of influenza B appearing earlier than the usual time interval may have been since we did not collect sufficient samples [19]. Harun and Beyza, while examining respiratory tract pathogens in the Bursa region, drew attention to the similarity between the frequency of influenza A and B [20].

## Conclusions

In conclusion, GAS pharyngitis and Influenza virus infection continue to be frequent etiologies of URTI in childhood. Our study revealed that viral influenza A and B infections in children under five years and GAS infection in children older than five years were more common in the Istanbul metropolitan area, consistent with current multicentric studies. It is essential to recognize influenza infections in under five years in terms of lower respiratory tract infections and other comorbidities that may require hospitalization. Over the years, recognizing influenza complaints and performing the necessary tests have helped clarify the diagnosis. More cases were identified by clarifying the diagnosis by performing more GAS and influenza screening tests by years, but the order of agents in URTI under five years of age and above did not change. The fact that the frequency of GAS infection does not change over the study periods indicates that GAS infection is still an important childhood infection. Most often, young children are infected by family members or siblings who go to school. The fact that a substantial portion of GAS infections occurs in under five years of age shows that rapid streptococcal screening can also help diagnose children with fever and accompanying lymphadenopathy and oropharyngeal hyperemia without flu symptoms.
